# An Atypical Pediatric Presentation of a Chronic Polyradiculoneuropathy

**DOI:** 10.7759/cureus.44361

**Published:** 2023-08-30

**Authors:** Wes Speer, Christopher Szewczyk, Ryan Jacobson

**Affiliations:** 1 Psychiatry, Rush Medical College, Chicago, USA; 2 Neurology, Rush Medical College, Chicago, USA; 3 Neurology, Rush University Medical Center, Chicago, USA

**Keywords:** albuminocytologic dissociation, areflexia, peripheral neuropathy, neuroimmunology, chronic inflammatory demyelinating polyneuropathy

## Abstract

Here, we present a case of a 15-year-old male with polyradiculoneuropathy, which was diagnosed as chronic inflammatory demyelinating polyneuropathy (CIDP), who was refractory to initial treatment. The patient presented with a one-and-a-half-month history of decreased strength, most notable in the bilateral hip flexors and finger flexors/extensors, and areflexia. Electromyography and nerve conduction studies did not fulfill diagnostic criteria for a demyelinating polyneuropathy; however, the cerebrospinal fluid analysis demonstrated albuminocytologic dissociation and his physical exam was otherwise consistent with the diagnosis. He was treated with IV immunoglobulin (IVIg). He relapsed less than one month later with worsening weakness. Imaging revealed increased cauda equina enhancement when compared to the MRI from the previous admission, and labs were otherwise similar to the initial presentation. He was treated with a second course of IVIg in addition to high-dose IV methylprednisolone. Upon his second discharge, he was transitioned to oral corticosteroids, and at a follow-up visit one month later, he had fully regained his strength and demonstrated normal reflexes. This case highlights the variable nature of CIDP in its initial presentation, its course, and its response to treatment, particularly in young patients. Additionally, we would like to emphasize that this case of CIDP was in the context of chronic malnutrition and significant weight loss, which made the diagnostic picture more complex.

## Introduction

Chronic inflammatory demyelinating polyneuropathy (CIDP) is an acquired, autoimmune condition characterized by demyelination of peripheral nerves resulting in progressive sensory loss and muscle weakness [1]. Epidemiologic data for CIDP are highly variable between regions of the world but the incidence seems to be estimated at approximately one to 10 per 100,000 and is higher in males than females [2]. Risk factors for CIDP include antecedent infections as well as certain dietary and lifestyle habits, such as smoking and alcohol consumption [3]. The underlying pathophysiology of CIDP is characterized by demyelination of peripheral nerves occurring both proximally and distally, and the weakness tends to have a symmetric distribution [4]. The mechanisms by which CIDP occurs have been described as autoimmune in nature, and while there is evidence to support cell-mediated and humoral immune system involvement, a specific etiology remains unclear [5].

The differential diagnosis includes Guillain-Barré syndrome (GBS), most commonly the acute inflammatory demyelinating polyneuropathy (AIDP) subtype. CIDP is differentiated from AIDP by a temporal cutoff of four weeks for AIDP and eight weeks for CIDP [6]. Between four and eight weeks is considered to be an intermediate zone that may be dubbed subacute inflammatory demyelinating polyneuropathy (SIDP) [7].

We would like to present an atypical pediatric case of polyradiculoneuropathy diagnosed as CIDP that occurred in the setting of malnutrition and an unspecified eating disorder that was initially refractory to treatment. This dysimmune neuropathy is only rarely observed in the pediatric population, which makes the diagnosis and treatment choices more challenging.

## Case presentation

A 15-year-old male without any significant past medical history presented with a one-and-a-half-month history of progressive muscle weakness most pronounced in the distal upper extremities and proximal lower extremities that progressed in severity and led to difficulty walking and falls. He reported a recent 35-pound (15.88 kg) weight loss via voluntary diet restriction over the course of four months prior to presentation. There was no recent viral illness or vaccination and no family history of any neurologic conditions.

Upon admission, the neurological exam was notable for diffuse weakness, worst in bilateral hip flexion, finger extension, and finger flexion. No sensory or autonomic disturbances were appreciated. There was no oral muscle involvement and deep tendon reflexes were absent. Laboratories assessing nutritional deficiencies were notable for borderline low folate, with a normal methylmalonic acid level. HIV, syphilis, and hepatitis B and C studies were also negative. Diagnostic studies were significant for magnetic resonance imaging (MRI) of the lumbar spine demonstrating mild enhancement of the cauda equina roots.

Nerve conduction studies (NCS) of the right upper extremity were performed, which did not demonstrate any sensory or motor abnormalities in the median or ulnar nerves. Electromyography (EMG) of the right deltoid, triceps brachii, and first dorsal interosseus also was normal. EMG and NCS in the lower extremity, which was clinically weak, demonstrated impersistent F-waves and mildly reduced recruitment activity in the deep peroneal nerve, suggesting neurogenic weakness. There were no other findings to suggest demyelination on this diagnostic testing despite his clinical manifestations. The sural sensory study was also normal. CSF analysis, which can be found in Table 1, was consistent with albuminocytologic dissociation. Given his symptomatology, physical exam, diagnostic data, and a time course of less than eight weeks, an immune neuropathy like AIDP was suspected. Thus, the patient was treated with a five-day course of 0.4 g/kg/day IV immunoglobulin (IVIg) for this presumptive diagnosis, in addition to nutritional supplementation (1 mg tablet folate daily continued through discharge) and physical therapy. His weakness improved with IVIg treatment from 3/5 to 4/5 most notably in the hip flexors, knee flexors, finger extensors, and finger flexors. Additionally, psychology was consulted due to symptoms of body dysmorphia, who advised outpatient therapy follow-up in addition to the dietician's recommendations. He was discharged to acute rehabilitation following the completion of treatment. Unfortunately, he developed worsening weakness within one month of starting inpatient physical rehabilitation and was readmitted for further evaluation.

**Table 1:** CSF data during initial admission

Test (units)	Reference range	Result
Albumin (mg/dL)	7-30	103
Glucose (mg/dL)	45-70	56
Protein (mg/dL)	7.0-35.0	130.2
CSF/serum albumin index	0-8	22
IgG (mg/dL)	0.0-5.2	8.1
WBC (/uL)	0-10	2
RBC (/uL)	0	1
% lymphocytes		94
% monocytes		6

Upon the second admission, the neurological exam was significant for weakness that was most pronounced in the distal upper extremities and proximal lower extremities, a pattern similar to when he was discharged. He remained areflexic and his sensory and cranial nerve exams were grossly normal. No involuntary movements were appreciated. Malabsorption labs showed slight abnormalities, which can be seen in Table 2. CSF studies (Table 3) and EMG/NCS were repeated, and an MRI of the brain and spinal cord was obtained.

**Table 2:** Malabsorption labs

Test (units)	Reference range	Result
Tissue transglutaminase antibody, IgG (U/mL)	0-5	6
Human leukocyte antigen (HLA)-DQ8	Negative	Positive
Fecal fat	Normal	Increased
Fecal calprotectin (ug/g)	0-120	41

**Table 3:** CSF data during the second admission

Test (units)	Reference range	Result
Glucose (mg/dL)	45-70	53
Protein (mg/dL)	7.0-35.0	134.0

**Figure 1:**
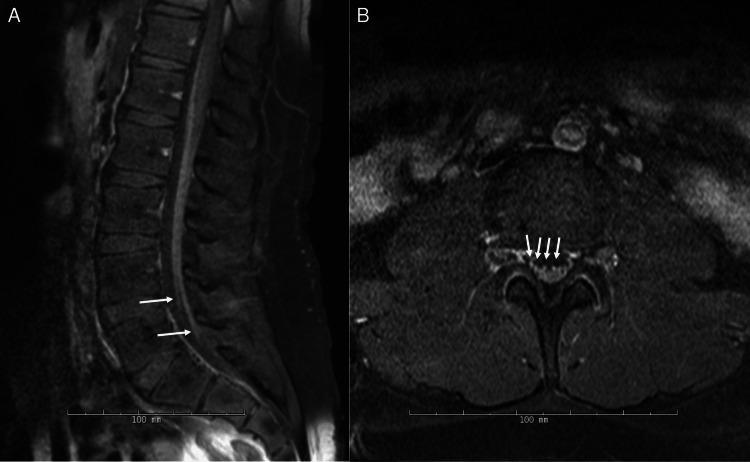
MRI of the lumbar spine from the second admission Image A (left): Postcontrast T1-weighted sagittal view demonstrating diffuse smooth enhancement of the cauda equina. Image B (right): Postcontrast T1-weighted axial view demonstrating enhancement of the cauda equina nerve roots.

EMG/NCS of the right upper and lower extremities again showed no clear demyelination, although F-waves remained absent or impersistent, showing increased latency. Amplitude and duration of compound muscle action potential were normal. Conduction velocities and latencies were normal except for slightly prolonged latency in the right peroneal nerve innervating the extensor digitorum brevis. Decreased recruitment was more clearly seen in clinically weak muscles, suggesting neurogenic weakness. MRI of the lumbar spine again showed enhancement of the cauda equina nerve roots, seen from a sagittal view in Figure 1A and an axial view in Figure 1B. He was re-treated with IVIg, and intravenous corticosteroids were added. Prior to discharge back to acute rehabilitation, he was transitioned to an oral prednisone taper with a plan to initiate maintenance IVIg treatments every three weeks. At the outpatient follow-up about a month later, the patient was on 20 mg oral prednisone daily and demonstrated marked improvement on the exam with full power and normal reflexes. Several months later, he remained improved, and a slow taper of both steroids and IVIg was continued.

## Discussion

This case of pediatric CIDP is striking in numerous ways and offers several important clinical reminders. First, the presentation itself was somewhat atypical. The patient’s clinical phenotype and course were consistent with CIDP, although he did not meet any electrodiagnostic criteria. Time to treatment was crucial for this patient and because repeat electrodiagnostic testing would not have otherwise changed management, it was not performed. Overall, CIDP is classically a polyradiculoneuropathy with dysfunction at both the level of the roots and peripheral nerves. Given his distribution of weakness, the lack of sensory deficits on physical exam, and no clear evidence of demyelination of sensory fibers on NCS, the dysfunction is predominantly proximal to the dorsal root ganglion, more specifically at the level of the nerve root. This is further supported by enhancement of the cauda equina seen on MRI with correlation of abnormal CSF findings. Although there is no genetic test for CIDP, certain inherited neuropathies can mimic its presentation and can be tested for (e.g., PMP22 gene mutation) [8]. In considering hereditary etiologies for this patient, due to the lack of familial history of neurologic disease, as well as no other findings that would lead us toward a genetic condition, such as abnormal foot arches, these causes were not on the initial differential.

An inflammatory demyelinating polyneuropathy is considered chronic when the disease has lasted longer than eight weeks and acute when the course has resolved in four weeks or less; a presentation between four and eight weeks is considered subacute. CIDP is differentiated from the acute and subacute presentations by a time course of eight weeks [9]. With regard to CIDP diagnostic criteria, the 2021 European Academy of Neurology and Peripheral Nerve Society criteria include clinical, laboratory, and electrodiagnostic components. The criteria describe CIDP as having one typical subtype and five variants. The typical subtype involves neuropathy with symmetrical proximal and distal weakness and sensory dysfunction that develop over the course of two months or more. The five variants are distal, multifocal, focal, pure motor, and pure sensory [1]. Each of these variants is based on clinical manifestations of the disorder rather than specific biomarkers.

To diagnose typical CIDP, the following clinical criteria must be met: progressive or relapsing, symmetric, proximal, and distal muscle weakness of upper and lower limbs, and sensory involvement of at least two limbs; development over at least eight weeks; and absent or reduced tendon reflexes in all limbs. All variants apart from pure motor must have sensory involvement. Thus, our patient, given that he had no measurable sensory deficits, may have fallen into the pure motor variant of CIDP. To diagnose pure motor CIDP, the following is required: clinical criteria, motor conduction criteria in two nerves, and normal sensory conduction in four nerves. If possible motor CIDP criteria are met and there is also a presence of at least two supportive criteria, pure motor CIDP can be diagnosed. To diagnose possible CIDP, motor conduction criteria in only one nerve is required in addition to the other motor CIDP criteria. This patient met the clinical criteria for pure motor CIDP, had two supportive criteria, with CSF findings and MRI, and had normal sensory conduction in the three nerves that were tested. Motor conduction criteria can be met in several ways with electrodiagnostic testing; however, this presentation was atypical even for the pure motor variant; there was no clear evidence of significant demyelination and no peripheral nerve slowing in the EMG/NCS studies. This could be due to his disease predominantly affecting the nerve roots, thus not allowing us to detect sensory abnormalities. With respect to imaging, there is currently no evidence to support the use of MRI in pediatric patients due to a lack of systematic studies, although it can be used to support the diagnosis of CIDP in adult patients who fulfill criteria for possible CIDP but not definite CIDP. Typical MRI findings supportive of CIDP are enlargement and/or increased signal intensity of nerve roots. MRI of this patient did show enhancement of the cauda equina roots, which is supportive of the diagnosis of CIDP but not diagnostic. In general, the role of neuroimaging in the diagnosis and treatment management of CIDP is an area that continues to be studied [10]. Patients such as the one presented in this case who do not otherwise fulfill diagnostic criteria present an opportunity for the use of neuroimaging [11]. Similarly, CSF analysis can also be considered in patients who fulfill criteria for possible CIDP, but not definite CIDP, as increased CSF protein, as seen in this case, is often present. Although the sensitivity and specificity are not particularly high for this test in CIDP, it is useful in excluding other diagnoses and supporting the diagnosis of CIDP, especially in cases of acute or subacute onset.

Second, the patient initially showed improvement with the first IVIg treatment but ultimately had a re-exacerbation of symptoms. IVIg is typically the first line for CIDP, including pure motor variants. Corticosteroids can also be used as first-line treatment, and if neither works, other treatment options like plasma exchange may be considered [1]. Other agents such as methotrexate, interferon beta 1a, and fingolimod are generally not recommended; however, some immunosuppressive drugs may be tried after the failure of other proven methods [1]. Understanding why he failed IVIg treatment is important because it can provide clues as to the specific variant of CIDP he had, which could elucidate the need for additional biomarkers in the diagnosis of CIDP to guide treatment decisions [12]. Specifically, IgG4 antibodies, such as anti-neurofascin-155, have been seen in CIDP patients refractory to IVIg, although these patients typically have different findings, including central nervous system symptoms [13]. Anti-contactin-1 antibodies have also been well described; however, CIDP in these patients tends to have prominent sensory ataxia [14].

In this case, the use of corticosteroids provided an additional treatment option that the patient was able to easily continue in the outpatient or rehabilitation setting [15]. Further, the patient may have had a relapsing-remitting type of CIDP, which is more common in children, though these patients tend to respond well to IVIg [16]. Overall, this points to a need for individualized treatment regimens, based on predictors of response to treatment, which could possibly be revealed through distinct CIDP subtype biomarkers [17]. This could help to prevent further clinical manifestations.

Third, the patient presented in the context of chronic malnutrition and significant weight loss. The relationship of these features to the development of CIDP in this case is unclear. Severe polyneuropathy may develop as a delayed complication in patients who have had bariatric surgeries, separate from measured vitamin or nutritional deficiencies. This suggests that rapid weight loss may trigger immune and inflammatory injury to neurons, which could potentially be of some relevance in this patient [18]. However, the time course in these case reports is more acute and describes some cases resembling GBS responding successfully to IVIg treatments [19].

Overall, more research is needed into the mechanisms of the development of CIDP, perhaps especially in the pediatric population. The variability in presentation and different responses to therapy suggest that there may be multiple factors at play in its pathogenesis. The use of additional biomarkers would be helpful in delineating various subtypes from one another, especially those that respond differently to treatment, which may improve treatment outcomes. Patients should be counseled about the nature of the disease process with treatment and followed to monitor its course.

## Conclusions

CIDP is an immune neuropathy that may present a significant diagnostic challenge to a clinician, especially if the presentation is atypical. This case was atypical as it affected a pediatric patient, which is rare and as such can lead to a delay in diagnosis. Further, the pathophysiology was predominantly that of an immune polyradiculopathy, which made the diagnosis more challenging electrodiagnostically.

Making an accurate and timely diagnosis of CIDP is important, as it can guide treatment decisions. However, the understanding of the association between CIDP subtypes and response to treatment remains incomplete. Further research is needed to identify the pathophysiologic underpinnings of this diagnosis, particularly in the pediatric realm, which could provide additional insight into treatment selection.
